# Supplementary material for: P-TRAP: a Panicle Trait Phenotyping tool
Source: BMC Plant Biol. 2013 Aug 29;13:122. doi: 10.1186/1471-2229-13-122 (PMC3848748; doi:10.1186/1471-2229-13-122)
Supplement: Additional file 7 — P-TRAP source code. The Netbeans project that contains the source code of the application. [file 1471-2229-13-122-S7.zip › javadoc.zip!/javadoc/org-bioalg-graphprocessor/org/bioalg/graphprocessor/Graph.html]

Graph (GraphProcessor)


JavaScript is disabled on your browser.


- Package
- Class
- Tree
- Deprecated
- Index
- Help

*org.bioalg.graphprocessor 1.0*

- Prev Class
- Next Class

- Frames
- No Frames

- All Classes

- Summary:
- Nested |
- Field |
- Constr |
- Method

- Detail:
- Field |
- Constr |
- Method


org.bioalg.graphprocessor

## Class Graph

- java.lang.Object
- - org.bioalg.graphprocessor.Graph

- ---

    

  ```
  public class Graph
  extends java.lang.Object
  ```

  The mathematical graph. The graph is directed from left to right.

  Author:
  :   faroq

- - ### Constructor Summary

    Constructors

    Constructor and Description

    `Graph()`
  - ### Method Summary

    Methods

    | Modifier and Type | Method and Description |
    | --- | --- |
    | `void` | `addEdge(Edge e)` |
    | `java.awt.Point` | `calclastGenereatingPoint()` Calculate the generating last generating point in the graph. |
    | `void` | `clear()` |
    | `Edge` | `getEdge(int i)` |
    | `java.util.ArrayList<Edge>` | `getEdges()` |
    | `java.awt.Point` | `getLastGenereatingPoint()` |
    | `int` | `getLength()` |
    | `java.util.ArrayList<java.awt.Point>` | `getMainbaranches()` |
    | `java.util.ArrayList<java.awt.Point>` | `getMainGeneratingPoints()` |
    | `java.util.ArrayList<java.awt.Point>` | `getSubGeneratingPoints()` |
    | `java.util.ArrayList<java.awt.Point>` | `getVertixNeighbors(java.awt.Point p)` Return the neighbors of a given point |
    | `boolean` | `hasEdge(Edge e)` Return true if the graph contains the input edge. |
    | `boolean` | `isGeneratingPoint(java.awt.Point p)` Return true if a given point has more than three neighbors |
    | `int` | `removeDuplicates(int threshold)` Remove duplicated vertices if any |
    | `boolean` | `removeEdge(Edge e)` |
    | `int` | `removeSpikes(int threshold)` Remove the spikes given a length threshold |
    | `int` | `removetranstions()` Remove the transition vertices from the graph. |
    | `void` | `setEdges(java.util.ArrayList<Edge> edges)` |
    | `GraphModel` | `toGraphModel()` Convert this graph to a graph model. |

    - ### Methods inherited from class java.lang.Object

      `clone, equals, finalize, getClass, hashCode, notify, notifyAll, toString, wait, wait, wait`

- - ### Constructor Detail


    - #### Graph

      ```
      public Graph()
      ```
  - ### Method Detail


    - #### getEdges

      ```
      public java.util.ArrayList<Edge> getEdges()
      ```


    - #### getEdge

      ```
      public Edge getEdge(int i)
      ```


    - #### clear

      ```
      public void clear()
      ```


    - #### isGeneratingPoint

      ```
      public boolean isGeneratingPoint(java.awt.Point p)
      ```

      Return true if a given point has more than three neighbors

      Parameters:
      :   `p` - the point

      Returns:


    - #### calclastGenereatingPoint

      ```
      public java.awt.Point calclastGenereatingPoint()
      ```

      Calculate the generating last generating point in the graph.

      Returns:
      :   a point


    - #### getVertixNeighbors

      ```
      public java.util.ArrayList<java.awt.Point> getVertixNeighbors(java.awt.Point p)
      ```

      Return the neighbors of a given point

      Parameters:
      :   `p` - the input point


    - #### setEdges

      ```
      public void setEdges(java.util.ArrayList<Edge> edges)
      ```


    - #### hasEdge

      ```
      public boolean hasEdge(Edge e)
      ```

      Return true if the graph contains the input edge.

      Parameters:
      :   `e` - the edge


    - #### addEdge

      ```
      public void addEdge(Edge e)
      ```


    - #### removeEdge

      ```
      public boolean removeEdge(Edge e)
      ```


    - #### getLength

      ```
      public int getLength()
      ```


    - #### getLastGenereatingPoint

      ```
      public java.awt.Point getLastGenereatingPoint()
      ```


    - #### getMainGeneratingPoints

      ```
      public java.util.ArrayList<java.awt.Point> getMainGeneratingPoints()
      ```


    - #### getMainbaranches

      ```
      public java.util.ArrayList<java.awt.Point> getMainbaranches()
      ```


    - #### getSubGeneratingPoints

      ```
      public java.util.ArrayList<java.awt.Point> getSubGeneratingPoints()
      ```


    - #### toGraphModel

      ```
      public GraphModel toGraphModel()
      ```

      Convert this graph to a graph model. The graph model is more suitable for
      input/output operations.

      Returns:
      :   a `GraphModel`


    - #### removetranstions

      ```
      public int removetranstions()
      ```

      Remove the transition vertices from the graph.


    - #### removeSpikes

      ```
      public int removeSpikes(int threshold)
      ```

      Remove the spikes given a length threshold

      Parameters:
      :   `threshold` -

      Returns:


    - #### removeDuplicates

      ```
      public int removeDuplicates(int threshold)
      ```

      Remove duplicated vertices if any

      Parameters:
      :   `threshold` - the minimum length two vertices are removed

      Returns:
      :   number of removed vertices


- Package
- Class
- Tree
- Deprecated
- Index
- Help

*org.bioalg.graphprocessor 1.0*

- Prev Class
- Next Class

- Frames
- No Frames

- All Classes

- Summary:
- Nested |
- Field |
- Constr |
- Method

- Detail:
- Field |
- Constr |
- Method
